# Decreased Cerebrospinal Fluid Flow Is Associated With Cognitive Deficit in Elderly Patients

**DOI:** 10.3389/fnagi.2019.00087

**Published:** 2019-04-30

**Authors:** Jadwiga Attier-Zmudka, Jean-Marie Sérot, Jeremy Valluy, Mo Saffarini, Anne-Sophie Macaret, Momar Diouf, Salif Dao, Youcef Douadi, Krzysztof Piotr Malinowski, Olivier Balédent

**Affiliations:** ^1^Department of Gerontology, Centre Hospitalier de Saint-Quentin, Saint-Quentin, France; ^2^CHIMERE, EA 7516 Head and Neck Research Group, University of Picardie Jules Verne, Amiens, France; ^3^ReSurg SA, Nyon, Switzerland; ^4^Department of Neurology, Centre Hospitalier de Saint-Quentin, Saint-Quentin, France; ^5^Department of Research, Amiens University Hospital, Amiens, France; ^6^Department of Radiology, Centre Hospitalier de Saint-Quentin, Saint-Quentin, France; ^7^Faculty of Health Sciences, Institute of Public Health, Jagiellonian University Medical College, Kraków, Poland; ^8^BioFlowImage, Image Processing Unit, University Hospital of Amiens, Amiens, France

**Keywords:** cerebrospinal fluid, cognitive dysfunction, aged patients, dementia, magnetic resonance imaging, phase contrast

## Abstract

**Background:** Disruptions in cerebrospinal fluid (CSF) flow during aging could compromise protein clearance from the brain and contribute to the etiology of Alzheimer’s Disease (AD).

**Objective:** To determine whether CSF flow is associated with cognitive deficit in elderly patients (>70 years).

**Methods:** We studied 92 patients admitted to our geriatric unit for non-acute reasons using phase-contrast magnetic resonance imaging (PC-MRI) to calculate their ventricular and spinal CSF flow, and assessed their global cognitive status, memory, executive functions, and praxis. Multivariable regressions with backward selection (criterion *p* < 0.15) were performed to determine associations between cognitive tests and ventricular and spinal CSF flow, adjusting for depression, anxiety, and cardiovascular risk factors.

**Results:** The cohort comprised 71 women (77%) and 21 (33%) men, aged 84.1 ± 5.2 years (range, 73–96). Net ventricular CSF flow was 52 ± 40 μL/cc (range, 0–210), and net spinal CSF flow was 500 ± 295 μL/cc (range, 0–1420). Ventricular CSF flow was associated with the number of BEC96 figures recognized (β = 0.18, CI, 0.02–0.33; *p* = 0.025). Spinal CSF flow was associated with the WAIS Digit Span Backward test (β = 0.06, CI, 0.01–0.12; *p* = 0.034), and categoric verbal fluency (β = 0.53, CI, 0.07–0.98; *p* = 0.024) and semantic verbal fluency (β = 0.55, CI, 0.07–1.02; *p* = 0.024).

**Conclusion:** Patients with lower CSF flow had significantly worse memory, visuo-constructive capacities, and verbal fluency. Alterations in CSF flow could contribute to some of the cognitive deficit observed in patients with AD. Diagnosis and treatment of CSF flow alterations in geriatric patients with neurocognitive disorders could contribute to the prevention of their cognitive decline.

## Introduction

The cerebrospinal fluid (CSF) is an important part of the central nervous system, as it allows exchange of water, small molecules and proteins between the brain parenchyma and arterial and venous blood (Oreskovic and Klarica, 2010; Brinker et al., 2014), by either passive diffusion or active transport (Oreskovic and Klarica, 2014; Oreskovic et al., 2017b). The CSF therefore plays an important role in regulating brain homeostasis, waste clearance (Puy et al., 2016), as well as intracranial pressure and blood supply (Baledent et al., 2004). During aging, CSF turnover can be disrupted (Rubenstein, 1998; Stoquart-ElSankari et al., 2007) which could contribute to the etiology of age-related neurocognitive disorders (Rubenstein, 1998; Weller et al., 2000; Launer, 2002; Silverberg et al., 2003; Chakravarty, 2004). Several studies revealed that patients with Alzheimer’s disease (AD) have disrupted CSF pressure (Silverberg et al., 2006), turnover (Henry-Feugeas and Intracranial, 2009; Serot et al., 2012), and oscillations (Silverberg et al., 2006; Stoquart-ElSankari et al., 2007). Moreover, biomarkers for AD are found in the CSF, and their abundance was shown to have predictive value for clinical progression (Wolfsgruber et al., 2017).

The increase of intracranial pressure during the cardiac cycle causes a flow from the blood and brain interstitial fluid to the CSF, and a net CSF flow toward its extracerebral compartment and venous blood (Oreskovic et al., 2017a). Since this CSF flow is important for protein clearance from the brain (Puy et al., 2016), it is possible that impaired CSF flow could be associated with cognitive decline (Coblentz et al., 1973; Sohn et al., 1973; Rubenstein, 1998). Moreover, CSF flow is linked with brain perfusion (Egnor et al., 2002; Baledent et al., 2004), defects of which are known causes of neurocognitive disorders in the elderly (O’Brien and Thomas, 2015). A number of studies suggested that the choroid plexus and the ventricular walls degenerate with the progression of AD (Serot et al., 2000; Balusu et al., 2016; Daouk et al., 2016), but none could determine whether disrupted CSF flow causes cognitive decline, or whether it is a by-product of AD or normal aging.

To the authors’ knowledge, there are no published studies that investigated the relationship between CSF flow alterations and cognitive deficit in the elderly, adjusting for cardiovascular risk factors for the development of neurocognitive disorders. The purpose of this study was therefore to evaluate the association of CSF flow in the brain ventricles and cervical spine with cognitive deficit (assessed using neurocognitive tests in clinical settings) in a cohort of elderly patients (>70 years) admitted to our geriatric unit for non-acute reasons. The hypothesis was that reduced CSF flow would be associated with cognitive deficit. Improved knowledge of such associations could guide the development of medical or surgical treatments to limit or prevent cognitive decline in the elderly (Nakajima et al., 2018).

## Materials and Methods

**FIGURE 1:**
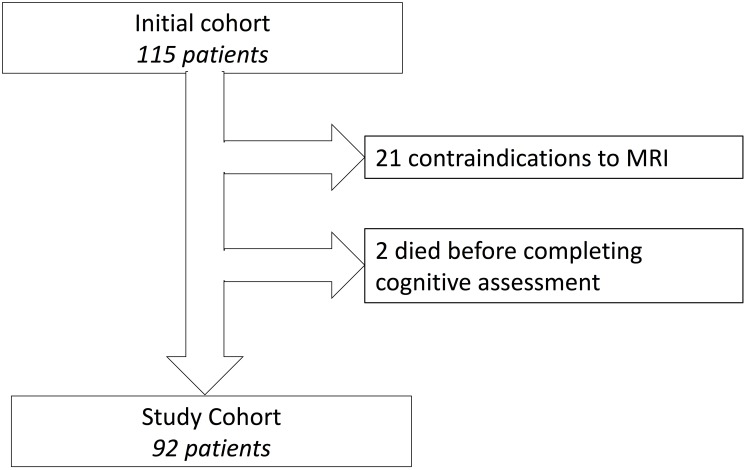
Flowchart of patient inclusion.

We enrolled 115 consecutive patients admitted to our geriatric unit for non-acute reasons between October 2015 and March 2018. The inclusion criterion was patients aged over 70 years. Twenty-one patients were excluded because of contraindications to phase-contrast magnetic resonance imaging (PC-MRI) for analysis of brain fluid motion, and two patients died before completing neurocognitive assessments. This left a study cohort of 92 patients (Figure 1) who underwent PC-MRI that enabled calculation of ventricular (aqueduct) CSF flow and spinal (C2-C3) CSF flow.

### Data Acquisition

The PC-MRI was performed using a 1.5-T machine. Conventional morphologic image sequences were first acquired in the sagittal and axial planes. The CSF flow acquisition planes were then selected perpendicular to the presumed direction of flow through the Sylvius aqueduct [representing the ventricular flow (Jacobson et al., 1996)] and the spinal C2-C3 sub-arachnoid spaces (representing the spinal flow). Flow images were acquired using a velocity-encoded phase-contrast pulse sequence with peripheral gating, as previously described (Baledent et al., 2001, 2004). Velocity sensitization was set at 10 cm/s for the ventricular flow and 5 cm/s for the spinal flow.

### Data Analysis

Phase-contrast magnetic resonance imaging data were transferred to a Sparc 10 workstation (SUN Microsystems) and analyzed using an in-house image processing software with Interactive Data Language (Baledent et al., 2001). This software automatically measures the CSF flow curve over the cardiac cycle for a given region of interest. Cranial–caudal flows were positive (CSF flush), whereas caudal–cranial flows were negative (CSF fill). The difference between CSF fill and flush flows is the net CSF flow, which reflects the volume of CSF produced (Nilsson et al., 1994). For technical reasons, intracranial sub-arachnoid CSF flow was not investigated.

### Neurocognitive Assessment

All patients underwent a battery of neurocognitive tests that assessed global cognitive status, memory, executive functions, praxis, as well as depression and anxiety (Table 2). Global cognitive efficiency was assessed by the Mini-Mental State Examination (MMSE) (Folstein et al., 1975), standard of Kalafat et al. (2003), following the GRECO standardization and calibration, and the Mattis Dementia Rating scale (MDRS) (Hersch, 1979; Gardner et al., 1981). The memory domain was assessed using the Wechsler Adult Intelligence Scale (WAIS-III) – Digit Span task (Iverson and Tulsky, 2003; Hill et al., 2010) and the Grober-Buschke (GB) test (French version of the Free and Cued Selective Reminding Test) (Buschke, 1984; Grober et al., 1988; Grober and Kawas, 1997; Van der Linden and Juillerat, 2004). Instrumental cognition was assessed with Signoret’s Battery of Cognitive Efficacy (BEC 96) (visuo-constructive subscale) (Jacus et al., 2001). Attention and executive domains were assessed using the Stroop Color and Words Test (Comalli et al., 1962), as well as two categoric and semantic verbal fluency tests, as impaired semantic fluency is a predictor of progression to AD (Vaughan et al., 2018) and categoric fluency may be impaired in amnesic MCI (Balthazar et al., 2007). In addition, the patients were evaluated with the Montgomery-Asberg Depression rating scale (MADRS) (Montgomery and Asberg, 1979), on 60 points, and the Goldberg anxiety scale (Goldberg et al., 1987; Huber et al., 1999) on 9 points, to rule out effects of depression or anxiety on cognitive test results.

Mild cognitive impairment was diagnosed based on the criteria of Petersen et al. (Petersen et al., 2001), while AD and AD-like diseases were diagnosed according to the Diagnostic and Statistical Manual of Mental Disorders, 4th Edition (DSM-IV) and the recommendations of the National Institute on Aging – Alzheimer’s Association workgroups (McKhann et al., 2011).

Vascular risk factors were diagnosed based on a comprehensive geriatric assessment performed at our geriatric unit. Blood samples were obtained after a minimum of 10 h of fasting. The diagnosis of diabetes was attributed with blood glucose levels above 7 mmol/L, anemia with hemoglobin levels below 12 (women) or 13 (men) g/dL and inflammation with CRP levels above 10 mg/L. Likewise, the reference range was 4.1–6.5 mmol/L for total cholesterol, 0.6–1.8 mmol/L for triglycerides and 35–50 g/L for Albumin. Malnutrition was defined by Albumin levels <35 g/L.

### Statistical Analysis

Descriptive statistics were used to summarize the data. Shapiro–Wilk tests were used to assess the normality of distributions. Pearson’s or Spearman’s coefficients were calculated to identify significant correlations between CSF flow (ventricular and spinal) and all cognitive test results. The ventricular CSF flow was correlated with the BEC96 visuo-constructive subscale (*r* = 0.231, *p* = 0.037), while the spinal CSF flow was correlated with the backward WAIS digit span task (*r* = 0.251, *p* = 0.023) and the GB recall test (*r* = 0.230, *p* = 0.054). Multivariable regressions with backward selection (criterion *p* < 0.15) were performed to determine associations between the aforementioned cognitive tests and thirteen independent variables, to adjust for depression, anxiety, and cardiovascular risk factors (Barnes and Yaffe, 2011) [gender, age, BMI, CSF flow (ventricular), CSF flow (spinal), MADRS, and Goldberg scores, as well as presence of diabetes, inflammation, hypercholesterolemia, hypertriglyceridemia, malnutrition or anemia]. Statistical analyses were performed using R version 3.3.2 (R Foundation for Statistical Computing, Vienna, Austria). *P*-values <0.05 were considered statistically significant.

## Results

The final cohort comprised 71 women (77%) and 21 (33%) men, aged 84.1 ± 5.2 years (range, 73–96), with BMI 25.2 ± 4.8 kg/m^2^ (range, 15.6–40.0) (Table 1). The MADRS score was 7.3 ± 6.6 (range, 0–31), and 4 patients (4.3%) had scores >20, indicating moderate to severe depression. The Goldberg score was 2.8 ± 2.4 (range, 0–9), and 23 patients (25%) had scores ≥5, indicating possible anxiety. Net ventricular CSF flow was calculated at 52 ± 40 μL/cc (range, 0–210), and net spinal CSF flow at 500 ± 295 μL/cc (range, 0–1420). The majority of patients reported cognitive impairment and overall diagnosis revealed mild cognitive impairment in 41 patients (46%), vascular dementia in 11 (12%), AD in 5 (6%), Lewy-body dementia in 1 (1%), and mixed dementia in 32 (36%). The cognitive assessment echoed these findings with an MMSE score of 22 ± 4.8 (range, 7–30), and a MDRS or 117.7 ± 17.8 (range, 60–143) (Table 2).

**Table 1:** Patient demographics.

	Cohort (*n* = 92)	
	mean ±*SD*	(range)
Women	71 (77%)	
Age (years)	84.1 ± 5.2	(73–96)
BMI (kg/m^2^)	25.2 ± 4.8	(15.6–40.0)
Depression (MADRS^a^/60)	7.3 ± 6.6	(0–31)
Anxiety (Goldberg/9)	2.8 ± 2.4	(0–9)
**CSF flow**		
Ventricular (μL/cc^b^)	52 ± 40	(0–210)
Spinal (μL/cc^b^)	500 ± 295	(0–1420)
Diabetes	11 (12%)	
Inflammation	40 (43%)	
Hypercholesterolemia	5 (5%)	
Hypertriglyceridemia	19 (21%)	
Malnutrition	65 (71%)	
Anemia	57 (62%)	
**Diagnosis**		
Mild cognitive impairment	41 (46%)	
Vascular dementia	11 (12%)	
Alzheimer’s disease	5 (6%)	
Dementia with Lewy bodies	1 (1%)	
Mixed dementia	32 (36%)	


*^a^MADRS, Montgomery-Asberg Depression Rating Scale; ^a^cc, cardiac cycle.*

**Table 2:** Cognitive assessment.

	Cohort (*n* = 92)	
	mean ±*SD*	(range)
**General Cognition**		
Mini Mental-State Exam (MMSE)	22.0 ± 4.8	(7–30)
Mattis Dementia Rating Scale (MDRS)	117.7 ± 17.8	(60–143)
**Memory Domain**		
Wechsler Adult Intelligence Scale - (WAIS) Digit Span task		
Forward (numbers)	4.4 ± 0.9	(3–7)
Backward (numbers)	2.9 ± 0.8	(2–6)
Grober-Bushke Free and Cued Selective Reminding Test		
Encoding (words)	13.8 ± 2.9	(3–16)
Recall (words)	13.8 ± 9.5	(0–36)
Storage (words)	32.8 ± 14.9	(0–48)
**Intrumental Cognition Domain**		
BEC96 visuo-constructive subscale (Figures)	8.5 ± 2.9	(0–12)
**Executive Domain**		
Stroop Color and Word Test		
Colors (delay, s)	23.4 ± 12.8	(6–70)
Colors (non-corrected mistakes)	0.3 ± 1.0	(0–6)
Words (delay, s)	42.1 ± 25.4	(14–147)
Words (non-corrected mistakes)	1.1 ± 2.1	(0–9)
Interference (delay, s)	55.9 ± 24.5	(15–116)
Interference (non-corrected mistakes)	8.3 ± 7.9	(0–24)
**Verbal Fluency Test**		
Semantics (words)	9.3 ± 6.5	(0.0–34.0)
Categories (words)	12.8 ± 6.6	(1.0–33.0)


Multivariable analysis of factors affecting the memory domain revealed that (i) the WAIS Digit Span Backward results improved with increasing spinal CSF flow (β = 0.06, CI, 0.01–0.12; *p* = 0.034) and were worse for patients with anemia (β = -0.54, CI, -0.90 to -0.18; *p* = 0.004); (ii) the GB recall results increased with spinal CSF flow (β = 0.70, CI, -0.01–1.14; *p* = 0.054) and were worse in patients with malnutrition (β = -6.30, CI, -10.88 to -1.73; *p* = 0.008) (Table 3).

**Table 3:** Multi-variable regressions to identify factors associated with the memory domain (Backward selection *p* < 0.15).

	WAIS Digit Span Backwards (*n* = 80)			GB recall (n = 70)		
	regression coefficient	95% C.I. (range)	*p*-value	regression coefficient	95% C.I. (range)	*p*-value
Male Gender						
Age (years)						
BMI (kg/m^2^)						
CSF Flow (Ventricular^a^)						
CSF Flow (Spinal^b^)	0.06	(0.01– 0.12)	0.034	0.70	(-0.01–1.41)	0.054
Depression (MADRS)	-0.02	(-0.04– 0.01)	0.162			
Anxiety (Goldberg)						
Diabetes						
Inflammation						
Hypercholesterolemia				-6.01	(–15.39– 3.36)	0.205
Hypertriglicerydemia						
Malnutrition				-6.30	(-10.88 to -1.73)	0.008
Anemia	-0.54	(-0.90 to -0.18)	0.004			


*^a^regression coefficients given for increments of 10 μL/cc. ^b^regression coefficients given for increments of 100 μL/cc.*

Multivariable analysis also revealed that (i) the number of BEC96 figures recognized increased with ventricular CSF flow (β = 0.18, CI, 0.02–0.33; *p* = 0.025) and decreased in patients with malnutrition (β = -1.48, CI, -2.81 to -0.15; *p* = 0.029) (Table 4); (ii) categoric verbal fluency increased with spinal CSF flow (β = 0.53, CI, 0.07–0.98; *p* = 0.024) and in men (β = 3.88, CI, 0.68–7.08; *p* = 0.018), and was decreased in patients with diabetes (β = -7.48, CI, -11.92 to -3.05; *p* = 0.001); (iii) semantic verbal fluency increased with spinal CSF flow (×100) (β = 0.55, CI, 0.07–1.02; *p* = 0.024) and was decreased in patients with diabetes (β = -5.08, CI, -9.66 to -0.51; *p* = 0.030).

**Table 4:** Multi-variable regressions to identify factors associated cognitive tests (Backward selection *p* < 0.15).

	BEC96 Figures (*n* = 78)			Verbal fluency (categoric) (*n* = 76)			Verbal fluency (semantic) (*n* = 76)		
	regression coefficient	95% C.I. (range)	*p*-value	regression coefficient	95% C.I. (range)	*p*-value	regression coefficient	95% C.I. (range)	*p*-value
Male Gender				3.88	(0.68–7.08)	0.018			
Age	
BMI	
CSF Flow (Ventricular^a^)	0.18	(0.02–0.33)	0.025						
CSF Flow (Spinal^a^)				0.53	(0.07–0.98)	0.024	0.55	(0.07– 1.02)	0.024
Depression (MADRS)									
Anxiety (Goldberg)									
Diabetes				-7.48	(-11.92 to -3.05)	0.001	-5.08	(-9.66 to -0.51)	0.030
Inflammation									
Hypercholesterolemia									
Hypertriglicerydemia									
Malnutrition	-1.48	(-2.81 to -0.15)	0.029	-2.58	(-5.43–0.27)	0.076			
Anemia									


*^a^regression coefficients given for increments of 10 μL/cc. ^b^regression coefficients given for increments of 100 μL/cc.*

## Discussion

The main finding of this study is that CSF flow was associated with cognitive test results. Ventricular CSF flow was associated with the number of recognized BEC 96 figures, while spinal CSF flow was associated with the WAIS digit-span backward test, categoric and semantic verbal fluencies, as well as the Grober-Buschke recall test (borderline). Taken together, these results suggest that CSF flow influences memory, visuo-constructive capacities, and verbal fluency. Interestingly, our findings are coherent with CSF flow values reported in recent literature. Our measures of ventricular CSF flow were comparable to values reported for healthy adults, but higher than those reported for healthy elderly volunteers, while our measures of spinal CSF flow were slightly higher than those reported for all elderly patients, except those with amnesic mild cognitive impairment (Table 5; Baledent et al., 2004; El Sankari et al., 2011; Puy et al., 2016).

**Table 5:** CSF flow measures in our cohort and reported in the recent literature.

Author	Date	Journal	Cohort	*n*	Ventricular CSF Flow (μL/cc)	Spinal CSF Flow (μL/cc)
	(year)				mean ±*SD*	mean ±*SD*
*This study*	*–*	*–*	*Hospitalized elderly*	92	52 ± 40	500 ± 295
Puy et al.	2016	Frontiers in Aging Neuroscience	*CH patients*	9	174 ± 174	444 ± 188
El Sankari et al.	2011	Fluids and Barriers of the CNS	*Healthy elderly*	12	34 ± 17	457 ± 154
El Sankari et al.	2011	Fluids and Barriers of the CNS	*aMCI patients*	10	73 ± 33	584 ± 152
El Sankari et al.	2011	Fluids and Barriers of the CNS	*AD patients*	9	39 ± 18	450 ± 221
El Sankari et al.	2011	Fluids and Barriers of the CNS	*NPH patients*	13	167 ± 89	455 ± 221
Balédent et al.	2004	Investigative Radiology	*Healthy adults*	16	51 ± 25	467 ± 147
Balédent et al.	2004	Investigative Radiology	*CH patients*	12	196 ± 100	467 ± 260


*cc, Cardiac Cycle; CH, Communicating Hydrocephalus; aMCI, amnesic mild cognitive impairment; AD, Alzheimer’s disease; NPH, normal-pressure hydrocephalus.*

It is unclear how CSF flow alterations could affect cognition. While CSF flow could be influenced by blood perfusion (Baledent et al., 2004), the cognitive picture of altered CSF flow offered in this study is more reminiscent of AD (Chakravarty, 2004) rather than vascular dementias, in which memory deficits are less constant (O’Brien and Thomas, 2015). Impaired CSF flow was shown to affect the local biochemical composition of CSF (Puy et al., 2016), and thus, could affect protein clearance. This could lead to amyloid or phosphorylated tau accumulation in the brain parenchyma, which are thought to be important elements in the etiology of AD, and whose concentration in the CSF was shown to predict clinical development (Wolfsgruber et al., 2017). This is consistent with ongoing research into CSF biology suggesting that CSF and interstitial fluid exchange along a brain-wide perivascular network, linking glial and vascular function (Simon and Iliff, 2016). This “glymphatic system” is hypothesized to contribute to neuroinflammation and neurodegeneration, especially in the aging brain. CSF flow disruptions are common in AD (Silverberg et al., 2006; Stoquart-ElSankari et al., 2007; Henry-Feugeas and Intracranial, 2009; Serot et al., 2012), confirming an etiological role for CSF flow disturbance in the development of late-onset AD.

Normal pressure hydrocephalus (NPH) is a rare form of neurocognitive disorder, in the elderly – characterized by the triad of dementia, gait apraxia and incontinence – thought to be caused by impaired CSF circulation (Leinonen et al., 2017). Its effects on cognition are similar to AD, and several studies (Del Bigio et al., 1997; Bech et al., 1999; Savolainen et al., 1999; Silverberg et al., 2006) underlined the high prevalence of AD in patients meeting clinical criteria for hydrocephalus. Results of hydrocephalus treatment by ventriculoperitoneal shunting are promising (Silverberg et al., 2002; Nakajima et al., 2018), but a trial to apply this technique in AD patients showed no benefits (Silverberg et al., 2008). Thus, it is likely that AD and NPH are separate disorders, with some clinical similarity. However, due to its rarity and to partial overlap of symptoms, NPH is often misdiagnosed as AD. Unlike in AD, where ventricular CSF flow is normal or decreased (Silverberg et al., 2006), in NPH, ventricular CSF flow is increased (El Sankari et al., 2011). Measurement of CSF flow as performed in this study thus presents the opportunity to better diagnose neurocognitive disorders of the elderly and offer appropriate treatment. Further, a proportion of AD patients is characterized by symptoms resembling NPH (Silverberg et al., 2002; Chakravarty, 2004), and in light of our findings, it is likely that CSF flow impairment is responsible for some cognitive impairment in these patients, who could therefore benefit from treatment (Attier-Zmudka et al., 2016).

In this study, multivariable analysis revealed negative associations between diabetes and executive functions (as indicated by the verbal fluency tests), between malnutrition and episodic memory and executive function (indicated by the GB recall test and the categoric verbal fluency test, respectively), and between anemia and working memory (indicated by the WAIS digit span backward test). In addition, men had greater categoric fluency, which could be explained by the fact that they were on average younger than the women (80 ± 4.8 vs. 85 ± 4.9 years, respectively). Our findings are consistent with the literature: Diabetes is an important predictor of cognitive decline in the elderly and is associated with deficits in attention and executive functions (Ryan et al., 2016). Malnutrition is a broad disorder, but folate levels have been associated with reduced episodic memory (Hassing et al., 1999). Similarly, low hemoglobin levels are known to affect memory in the elderly (Shah et al., 2009).

This study is the first to report direct associations between CSF flow and cognition, and could improve our understanding of the complex etiologies and symptoms of neurocognitive disorders in the elderly. It shows that altered CSF flow is associated with cognitive deficits in elderly patients, and thus suggests that treatments aiming at restoring normal CSF flow could reduce cognitive deficits. This is in accordance with a clinical observation in our practice, of a patient who recovered from debilitating neurocognitive disorders after sleep apnea treatment and ventriculoperitoneal shunting (Attier-Zmudka et al., 2016). However, this study has certain limitations. First, hydrocephalus diagnosis could not be performed in these patients, though it was suspected in several cases. Advanced hydrocephalus presents a complex etiological picture, and the presence of these patients in our cohort could affect the results of the study. Second, patients with more severe cognitive deficit had difficulty in performing the tests, and could not provide data, so that the multivariable models do not make use of the full cohort. Third, the scope of the study is limited by the lack of control group of age-matched patients with no cognitive impairment, so we cannot extrapolate our findings to the general population. Finally, our list of competing risk factors was incomplete, and we were unable to correctly or systematically assess the patients’ smoking habits, alcohol consumption, or lack of physical activity.

In conclusion, this study revealed direct associations of ventricular and spinal CSF flows with cognitive scores in a geriatric population with cognitive impairment. CSF flow was associated with memory, visuo-constructive ability and verbal fluency. It is therefore possible that CSF flow alterations are responsible for at least a part of the cognitive deficit observed in our patients. Better diagnosis and treatment of CSF flow alterations in geriatric patients suffering from neurocognitive disorders is therefore recommended.

## Ethics Statement

Written informed consent was obtained from all patients for their participation and confirmed by their next-of-kin if necessary. The study protocol was approved by an independent Ethical Review Board (CPP Amiens: 2015/6) and the National Data Protection Authority (CNIL:150075B-31). The study was registered in clinicaltrials.gov (NCT02578303). All procedures were performed according to the Declaration of Helsinki.

## Author Contributions

JA-Z and OB designed the study, collected the data, and edited the manuscript. J-MS designed the study and edited the manuscript. JV wrote and edited the manuscript and performed the statistics. MS wrote and edited the manuscript. A-SM, SD, and YD collected the data. MD and KM validated the statistics.

## Conflict of Interest Statement

MS and JV are employed by ReSurg SA (research consulting firm that was paid by St Quentin Hospital to perform statistical analyses and write the manuscript). The remaining authors declare that the research was conducted in the absence of any commercial or financial relationships that could be construed as a potential conflict of interest.
